# Immigration projects among young doctors in Tunisia: Prevalence, destinations and causes

**DOI:** 10.1192/j.eurpsy.2022.1624

**Published:** 2022-09-01

**Authors:** M. Ajmi, M. Kahloul, I. Kacem, A. Chouchane, S. Ben Mansour, Y. Slama, M. Hafsia, M. Maoua, N. Mrizak, W. Naija

**Affiliations:** 1 Sahloul Academic Hospital, University of medicine, “Ibn Al Jazzar”, Sousse, Tunisia, Department Of Anesthesia And Intensive Care,, Sousse, Tunisia; 2 Farhat Hached Academic Hospital, Occupational Medicine, Sousse, Tunisia; 3 Sahloul Academic Hospital, 1- department Of Occupational Medicine, sousse, Tunisia

**Keywords:** causes, immigration, young doctors

## Abstract

**Introduction:**

The shortage of doctors has become a worrying problem in Tunisia. It is influenced by the phenomenon of immigration which remains poorly studied despite its magnitude.

**Objectives:**

To describe the migration intentions of Tunisian young doctors and to identify the associated factors that influence their decisions.

**Methods:**

This is a cross-sectional, analytical survey conducted between January and June 2019. It included all young doctors practicing in academic hospitals of Sousse (Tunisia). Data collection was based on a standardized self-administered questionnaire.

**Results:**

A total of 182 valid questionnaires were collected. The median age was 26.9±2.5 years and the sex-ratio was 0.47. Immigration projects were reported by 38.5% of participants. The main destination was France (36.3%%). The main contributing factors were marital status (p<10-3), resident status (p=0.002), surgical specialty (p<10-3), personal dissatisfaction (p=0.003), underpayment (p<10-3), workload and difficult work conditions (p<10-3), lack of appropriate training (p<10-3), financial crisis and economic instability (p<10-3), lack of a clear strategy for the healthcare system (p=0.005) and the impression by the model of other doctors who left Tunisia (p=0.01).

**Conclusions:**

The rate of migration intentions expressed in this study highlights the emergent need of interventions emanating from the Tunisian health-care system’s problems in order to stop the flow of young doctors towards developed countries in quest of better conditions.

**Disclosure:**

No significant relationships.

